# Cryptic diversity and virulence of *Beauveria bassiana* recovered from *Lycorma delicatula* (spotted lanternfly) in eastern Pennsylvania

**DOI:** 10.3389/finsc.2023.1127682

**Published:** 2023-04-25

**Authors:** Eric H. Clifton, Louela A. Castrillo, Stefan T. Jaronski, Ann E. Hajek

**Affiliations:** ^1^ Department of Entomology, Cornell University, Ithaca, NY, United States; ^2^ Emerging Pests and Pathogens Research, USDA-Agricultural Research Service, Ithaca, NY, United States; ^3^ Jaronski Mycological Consulting LLC, Blacksburg, VA, United States

**Keywords:** Beauveria, Lycorma delicatula, entomopathogenic fungi, planthopper, invasive insect

## Abstract

The entomopathogenic fungus *Beauveria bassiana* is cosmopolitan and known to infect a variety of sap-sucking pests like aphids, mealybugs, and scales in the order of Hemiptera. In Fall 2017, spotted lanternfly (SLF) adults killed by the fungal entomopathogen *B. bassiana* were found in Berks County, Pennsylvania. In 2018-2020 we collected SLF and nearby non-target insects killed by *Beauveria* spp. from 18 field sites in southeastern Pennsylvania. We identified 159 *Beauveria* isolates from SLF and six isolates from non-targets. Five isolates of *B. bassiana* and one isolate of *B. brongniartii* were identified from the non-targets. Based on sequence data from the nuclear B locus (Bloc) intergenic region, all the isolates from SLF were identified as *B. bassiana*, but there were 20 different strains within this species, grouped into two clades. Three *B. bassiana* strains (A, B, and L) were found in most field sites and were the most prevalent. Representative isolates for these three strains were used in laboratory bioassays and were compared to a commercial *B. bassiana* strain (GHA). Strain B was inferior to A, L, and GHA against nymphs; strains A and L had greater efficacy than B and GHA against adults. We also quantified conidial production on SLF cadavers. This paper discusses the diversity of these *B. bassiana* strains in SLF populations and implications for biological control of this abundant invasive.

## Introduction

Entomopathogenic fungi infect a diversity of insects but are well known as acute pathogens of hemipterans, including aphids, leafhoppers and planthoppers (1, 2). *Beauveria bassiana* (Bals.-Criv.) Vuill. (Hypocreales: Cordycipitaceae) is a well-studied entomopathogen (3, 4) and has been reported causing epizootics (sometimes as part of a complex of entomopathogens) in hemipteran pests, including the chinch bug (*Blissus leucopterus* (Say) [Hemiptera: Blissidae]) (5), elongate hemlock scale (*Fiorinia externa* (Ferris) [Hemiptera: Diaspididae]) (6), and kudzu bug (*Megacopta cribraria (Fabricius*) [Hemiptera: Plataspidae]) (7). In 2019, a co-epizootic caused by two native fungal pathogens, *B. bassiana* and *Batkoa major* (Thaxt.) Humber (Entomophthorales: Entomophthoraceae), was reported in two populations of the new invasive planthopper, the spotted lanternfly (SLF), *Lycorma delicatula* (White) (Hemiptera: Fulgoridae), in southeastern Pennsylvania (8). Clifton et al. (9) discovered two more species of hypocrealean fungi infecting SLF that are assumed to be native: *Metarhizium pemphigi* (Driver & R.J. Milner) (Hypocreales: Clavicipitaceae) and the new species *Ophiocordyceps delicatula* (Hypocreales: Ophiocordycipitaceae).

The genus *Beauveria* includes more than 12 cryptic species that cannot be identified with morphological characters alone (10–12). *Beauveria* isolates can be identified to species by sequencing the nuclear B locus (Bloc) intergenic region and other genes (12, 13). Previous studies describing *Beauveria* isolates that naturally infected invasives like the emerald ash borer (*Agrilus planipennis* Fairmaire [Coleoptera: Buprestidae]) (14) and coffee berry borer (*Hypothenemus hampei* (Ferrari) [Coleoptera: Curculionidae]) (13, 15) discovered multiple cryptic species and a wide assemblage of *B. bassiana* strains. Additional studies on these invasives involved bioassays and demonstrated how some native isolates of *B. bassiana* had greater virulence than a commercialized strain (GHA) and produced more conidia on their cadavers, which is indicative of greater epizootic potential (14, 16).

SLF is a new invasive, univoltine planthopper that was first discovered in Berks County, Pennsylvania in 2014 (17) and has spread to 13 additional US states (18). The native range of SLF includes China, Taiwan, and Vietnam, and SLF can be a sporadic pest in China feeding on tree of heaven, *Ailanthus altissima* (Mill.) Swingle (Sapindales: Simaroubaceae) (19, 20). SLF prefers tree of heaven which is invasive in North America, but will feed on wild and cultivated grapes (*Vitis* spp. [Vitales: Vitaceae]) as well as other woody plants; this species is now infamous for its voracious feeding, which has caused reduced productivity and mortality in grapevines (21–23). These impacts, as well as others on trees such as black walnut, *Juglans nigra* L. (Fagales: Juglandaceae) and red maple, *Acer rubrum* L. (Sapindales: Sapindaceae) (24–26), have resulted in the need for means of controlling this harmful insect. Eradication of SLF seems unlikely and long-term management tools, including natural enemies like entomopathogenic microorganisms, could help control this invasive species (27–29).

Mycoinsecticides containing different *B. bassiana* strains are available for commercial use in the United States (30). In field trial applications, BoteGHA (Certis USA; containing *B. bassiana* strain GHA) killed 43-48% of SLF nymphs and adults infesting *A. altissima* in a public park (31). Laboratory bioassays testing mycoinsecticides found that *Cordyceps javanica* (Frieder. & Bally) Kepler, B. Shrestha & Spatafora (Hypocreales: Cordycipitaceae) was less effective than *B. bassiana*, but all *B. bassiana*-based products had similar efficacy against SLF of different ages (32).

In 2018-2020 we conducted parallel studies on the genetic diversity of naturally occurring *B. bassiana* (this study) and *B. major* (33) that infect SLF. The goals of the current study were to (1) isolate and identify *Beauveria* spp. infecting SLF and non-targets, predominantly collected in natural forested areas and edge habitats (e.g., tree lines in a neighborhood), in southeastern Pennsylvania, and (2) describe the prevalence and distribution of indigenous *B. bassiana* strains in these areas invaded by SLF. This study was used to select indigenous *B. bassiana* strains for laboratory bioassays carried out in 2021 with potential for use in biological control of this new invasive pest.

## Materials and methods

### Sample collection

We collected fungal isolates from dead SLF or non-targets, mostly on the ground and at bases of trees, in Pennsylvania, USA. The first *B. bassiana* sample in this study was sent to our laboratory in August 2017 (site a, **Table 1**; **Figure 2**). In May 2018, we found four SLF adult cadavers with *B. bassiana* outgrowth beneath leaf litter in site b (**Table 1**; **Figure 2**). Although these SLF adults died in Fall 2017, we isolated these *B. bassiana* samples in 2018. In 2018-2020, we opportunistically sampled *Beauveria* spp. associated with SLF and non-target insects in 17 more sites among 5 counties in southeastern Pennsylvania (**Table 1**; **Figure 2**). Most of the sampling occurred in Berks County, Pennsylvania. Dense populations of SLF were mostly restricted to municipalities in and around eastern Berks County in 2014-2016. We only sampled sites around Lancaster and Philadelphia in 2020, mainly because SLF populations had only recently established there, and the Pennsylvania counties with these sites were added to the SLF quarantine in 2018 (34).

**Table 1:** Sampling sites in southeastern Pennsylvania from 2017 to 2020.

Map code	Site name	Pennsylvania County	Coordinates^a^	Elevation (meters)	Sampling year (# isolates)^b^
a	Boyertown Reservoir	Berks	40°20'25.2"N 75°41'00.9"W	184	2017 (1)
b	Fleetwood residence^a^	Berks	40°27'14.5"N 75°49'05.7"W	170	2017 (4); 2018 (1)
c	Angora Fruit Farm	Berks	40°21'30.9"N 75°52'59.9"W	214	2018 (31); 2019 (3); 2020 (6)
d	Blandon	Berks	40°26'31.4"N 75°52'54.3"W	114	2018 (2); 2019 (5); 2020 (4)
e	Conrad Road residence^a^	Berks	40°26'50.7"N 75°37'20.3"W	317	2018 (13); 2020 (8)
f	Kutztown University	Berks	40°30'32.8"N 75°46'29.9"W	138	2018 (9)
g	Lilitz residence^a^	Lancaster	40°09'26.0"N 76°18'26.0"W	117	2018 (1)
h	Penn State Berks	Berks	40°21'35.6"N 75°58'34.2"W	82	2018 (1)
i	Pottstown Quarry	Montgomery	40°14'07.9"N 75°35'25.8"W	70	2018 (12)
j	Schuler Road^a^	Berks	40°29'37.9"N 75°49'00.2"W	116	2018 (18); 2020 (1)
k	Leesport^a^	Berks	40°26'55.7"N 75°57'51.3"W	84	2019 (5)
l	Sinking Spring	Berks	40°19'36.0"N 76°02'21.9"W	116	2019 (14); 2020 (5)
m	Graffa Pond^a^	Lancaster	40°01'36.3"N 76°14'28.9"W	110	2020 (7)
n	Hill road	Berks	40°21'11.8"N 75°52'32.9"W	225	2020 (3)
o	Lancaster Central Park	Lancaster	40°01'13.5"N 76°16'42.5"W	114	2020 (2)
p	Overlook Park	Lancaster	40°05'00.1"N 76°19'11.2"W	108	2020 (6)
q	Schuylkill Center for Environmental Education	Philadelphia	40°03'38.1"N 75°14'44.5"W	113	2020 (1)
r	Treichlers Bridge	Lehigh	40°44'03.2"N 75°32'21.5"W	102	2020 (1)

a For sites located on private properties, coordinates are provided for nearby towns and intersections. Elevations for the areas of sampling are based on USGS data (https://apps.nationalmap.gov/viewer/). Sampling sites are marked on the map (see **Figure 1**).

b The number of isolates includes Beauveria spp. from non-targets.

For most of the *Beauveria*-infected SLF and non-target insects, we collected cadavers that already had fungal outgrowth that is characteristic for *Beauveria* spp. We identified non-target insects to family, genus, or species with a dissecting microscope following dichotomous keys for morphological characters. In some cases, we obtained isolates from SLF or non-target cadavers that had no fungal outgrowth at the time of sampling but later produced conidia after incubation on 1.5% water agar for 5-10 days in the laboratory. For non-target insects that already had fungal outgrowth at the time of sampling, we first isolated the fungus on selective media (see next section) before cleaning the cadaver with ethanol for identification. We found most of the cadavers in 2018-2020 near the bases of *A. altissima* trees (**Figure 1A**). In rare instances we found SLF adults with fungal outgrowth still attached to host trees by their mouthparts and/or legs (**Figure 1B**). In some sampling sites (e.g., Leesport and Sinking Spring) we collected live SLF and reared individuals on potted plants in a quarantine lab (for other studies), and some of these SLF later died from naturally occurring infections (i.e., these individuals were already infected with fungi before the time of sampling and rearing in the laboratory; see **Supplementary Materials**). Collaborators from USDA-APHIS (Kelly Murman, Stefani Cannon, Miriam Cooperband, John Baker, Regina Whitfield), Pennsylvania Department of Agriculture (Emily Fricke, Albert Ciccarone, Betsy Myers, Sandie Conway), and Penn State University (Emelie Swackhamer & Julie Urban) also helped with sampling SLF cadavers with fungal outgrowth in some sites outside of Berks County (e.g., Lancaster and Philadelphia).

**Figure 1:**
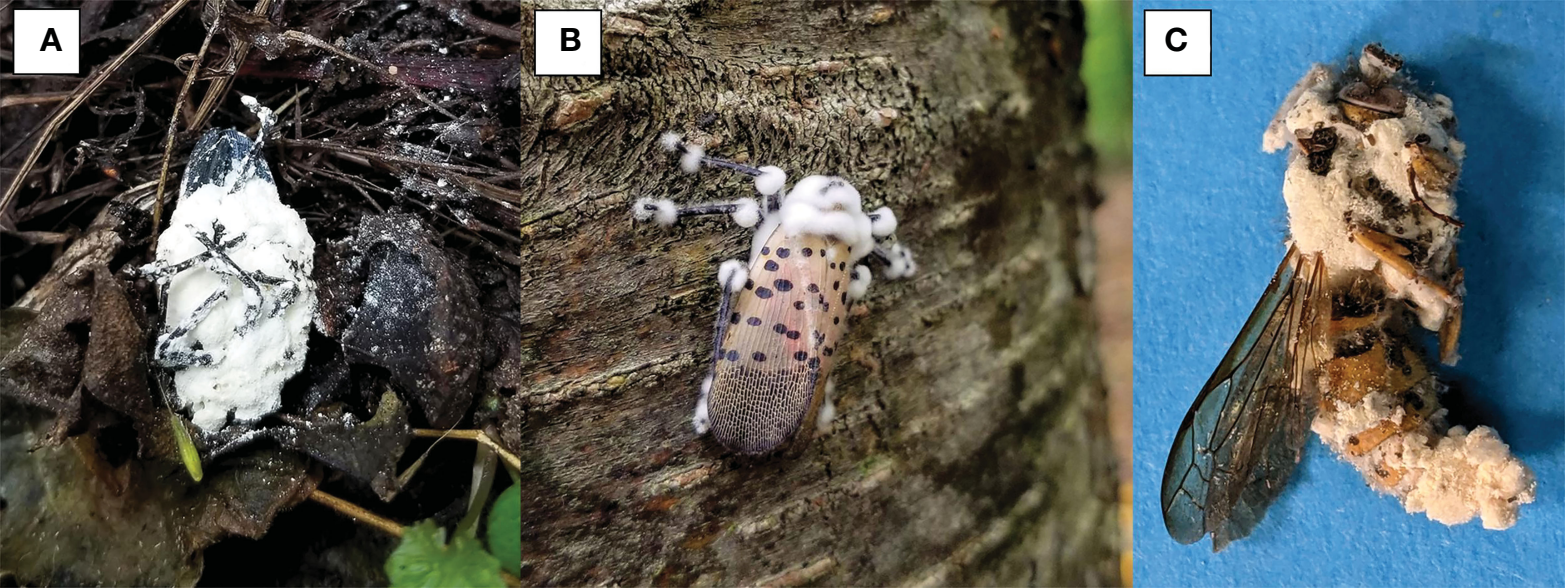
Examples of spotted lanternfly and a non-target with *Beauveria* fungal outgrowth. **(A)** Spotted lanternfly adult with profuse *Beauveria* outgrowth and conidia (infective spores). The white powdery spores are visible on the nearby ground and debris. **(B)** Spotted lanternfly adult killed by *Beauveria* and still attached to tree bark. **(C)** Non-target yellowjacket wasp with *Beauveria* outgrowth (later identified as *B brongniartii*).

### Isolation of fungi, DNA extraction, PCR, and sequencing

We swabbed conidia from SLF cadavers with fungal outgrowth with a sterile cotton-tip applicator and transferred to 6-cm Petri dishes containing selective media for *Beauveria*. The medium was adapted from Chase et al. (35), with 30 g wheat germ liter^−1^, autoclaved and then filtered through cheesecloth, before adding 0.25 g liter^−1^ chloramphenicol, 0.20 g dodine liter^−1^, 0.01 g crystal violet liter^−1^, and 15 g agar liter^−1^ before autoclaving again. We sealed selective media plates with Parafilm strips and placed in an incubator (20°C, 0:24 (L:D) h). After 10-14 days, we stored cultures of *Beauveria* isolates at 4°C until further use. Additional cultures of each *Beauveria* isolate were stored in 10% sterile glycerol at -80 °C in 2 mL cryovials (Nalgene, Thermo Fisher Scientific, Rochester, NY, USA).

We produced mycelium for DNA extraction from each *Beauveria* isolate following the protocol described in Clifton et al. (31) using potato dextrose broth. Bidirectional nucleotide sequences were determined for the nuclear Bloc intergenic region. A region of Bloc was amplified and sequenced using the primer pair B22-deg × B3.3R, following PCR conditions described by Rehner et al. (12). We used the SAP/EXO protocol to clean up reaction mixtures prior to sequencing (33). Sequencing was done by Cornell University Institute of Biotechnology on an ABI 3730x1 or through submissions to GeneWiz (https://www.genewiz.com/).

### *Beauveria* phylogenetics and prevalence of strains

We edited/trimmed, assembled, and aligned chromatograms and sequences with Geneious Prime software (2021.1.1; Biomatters Ltd.), resulting in contigs with 931-934 base pairs for the Bloc region. Sequence data were checked with the National Center for Biotechnology Information (NCBI) nucleotide database (https://blast.ncbi.nlm.nih.gov/Blast.cgi). After we identified unique strains of *B. bassiana*, we included the sequence data of each representative strain in the analysis, with one strain of *B. brongniartii* as an outgroup (**Table 2**). Maximum Likelihood (ML) analysis was performed using the rapid bootstrap algorithm in RAxML-HPC2 on XEDE version 8.1.11 using the default GTR+G in CIPRES Science Gateway online system (36). The Best Tree from ML analysis was drawn using FigTree version 1.4.4 (http://tree.bio.ed.ac.uk/software/figtree/). We deposited representative isolates of each strain with the USDA ARS Collection of Entomopathogenic Fungal Cultures (ARSEF, Ithaca, NY). ARSEF accession numbers and Genbank accession numbers are listed in **Table 2**.

**Table 2:** GenBank accession numbers of *Beauveria* spp. strains from spotted lanternfly (SLF) and other insect hosts analyzed in this study.

Representative strain (lab code; ARSEF accession no.)	Host(s) and location	B locus Genbank accession no.	Reference
B. bassiana			
A (18-02); ARSEF 14462	SLF (Pennsylvania, USA)	OP897311	This study
B (18-58); ARSEF 14463	SLF (Pennsylvania, USA)	OP897312	This study
C (18-57); ARSEF 14464	SLF (Pennsylvania, USA)	OP897313	This study
D (18-376); ARSEF 14465	Nitidulid beetle*	OP897314	This study
	(Coleoptera: Nitidulidae) (Pennsylvania, USA)		
E (18-56); ARSEF 14466	SLF (Pennsylvania, USA)	OP897315	This study
F (18-391); ARSEF 14467	SLF (Pennsylvania, USA)	OP897316	This study
G (18-328); ARSEF 14468	SLF (Pennsylvania, USA)	OP897317	This study
H (18-432); ARSEF 14469	SLF (Pennsylvania, USA)	OP897318	This study
I (18-427); ARSEF 14470	SLF (Pennsylvania, USA)	OP897319	This study
J (18-285); ARSEF 14471	SLF (Pennsylvania, USA)	OP897320	This study
K (18-333); ARSEF 14472	SLF (Pennsylvania, USA)	OP897321	This study
L (18-21); ARSEF 14473	SLF (Pennsylvania, USA)	OP897322	This study
M (18-65); ARSEF 14474	SLF (Pennsylvania, USA)	OP897323	This study
N (19-503); ARSEF 14475	SLF (Pennsylvania, USA)	OP897324	This study
O (18-63); ARSEF 14476	SLF (Pennsylvania, USA)	OP897325	This study
Q (18-393); ARSEF 14477	SLF (Pennsylvania, USA)	OP897326	This study
S (18-93); ARSEF 14478	SLF (Pennsylvania, USA)	OP897327	This study
T (18-329); ARSEF 14479	SLF (Pennsylvania, USA)	OP897328	This study
V (18-356); ARSEF 14481	Acalypterate fly* (Diptera) (Pennsylvania, USA)	OP897330	This study
Y (19-483); ARSEF 14482	SLF (Pennsylvania, USA)	OP897331	This study
GHA (commercial strain)	N/A (not applicable)	MN551319	Castrillo et al., (13)
Naturalis (commercial strain)	N/A (not applicable)	KM031766	N/A
ARSEF 1831	*Atta* sp.	DQ384380	Rehner et al., (11)
	(Hymenoptera) (Brazil)		
ARSEF 1853	*Dendroctonus ponderosae*	KM031773	Johny et al., (14)
	(Coleoptera) (Canada)		
ARSEF 4093	*Nezara viridula*	DQ384387	Rehner et al., (11)
	(Hemiptera) (Brazil)		
ARSEF 7972	(Coleoptera – unknown beetle)	KM031774	Johny et al., (14)
	(British Columbia, Canada)		
ARSEF 8170	*Agrilus planipennis*	KM031776	Johny et al., (14)
	(Coleoptera) (Michigan, USA)		
B. brongniartii (outgroup)			
(19-508); ARSEF 14483	*Vespula* sp.	OP897332	This study
	(Pennsylvania, USA)		
ARSEF 7376	*Magicicada septendecim*	HQ880701	Rehner et al., (12)

*The same B. bassiana strains recovered from these hosts have also been recovered from spotted lanternfly in the current study.

### *Beauveria bassiana* bioassays for *L. delicatula* nymphs and adults

#### Selection of *B. bassiana* isolates and inoculum production

Based on results from the field studies with *B. bassiana* strains, in early 2021 we chose six isolates to evaluate conidial production using biphasic-solid fermentation on flaked barley following the methods described by Jaronski & Jackson (37) (**Table 3**). These six isolates were representative of the prevalent strains (A, B, and L based on Bloc sequence data) in field sites with epizootics that occurred in 2018 (**Tables 1**; **3**).

**Table 3:** *Beauveria bassiana* isolates from adult spotted lanternfly that were used for solid substrate fermentation on barley flake.

Isolate #	Bloc strain	Collection site (2018)	Conidia yield (conidia/Kg substrate)	Standard deviation	Viability
02	A	Conrad Road residence	1.80 × 10^13^	4.39 × 10^12^	95%
45	A	Angora Fruit Farm	1.33 × 10^13^	2.00 × 10^12^	94%
03	B	Conrad Road residence	7.10 × 10^12^	1.81 × 10^11^	95%
58	B	Angora Fruit Farm	9.13 × 10^12^	2.02 × 10^12^	91%
21	L	Conrad Road residence	2.18 × 10^13^	4.05 × 10^12^	92%
67	L	Angora Fruit Farm	1.12 × 10^13^	1.92 × 10^12^	94%
GHA*	N/A	N/A	1.29 × 10^13^	1.43 × 10^12^	98%

**Figure 2:**
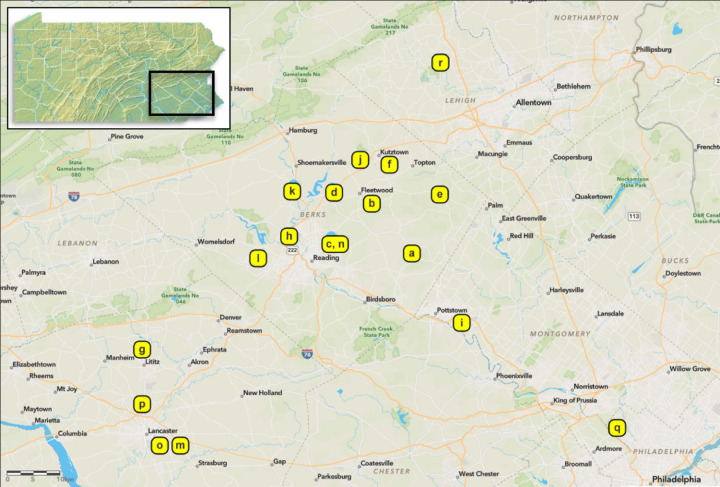
Sampling sites in southeastern Pennsylvania, USA. Inset image on the top left highlights the region. Isolates of *Beauveria* spp. were obtained from spotted lanternfly and non-targets in 2017 to 2020 (see **Table 1** for sampling site information). All sampling sites were infested with tree-of-heaven (*Ailanthus altissima*) and other host plants. More information about isolates and sampling methods are provided in the **Supplementary Materials**.

**Figure 3:**
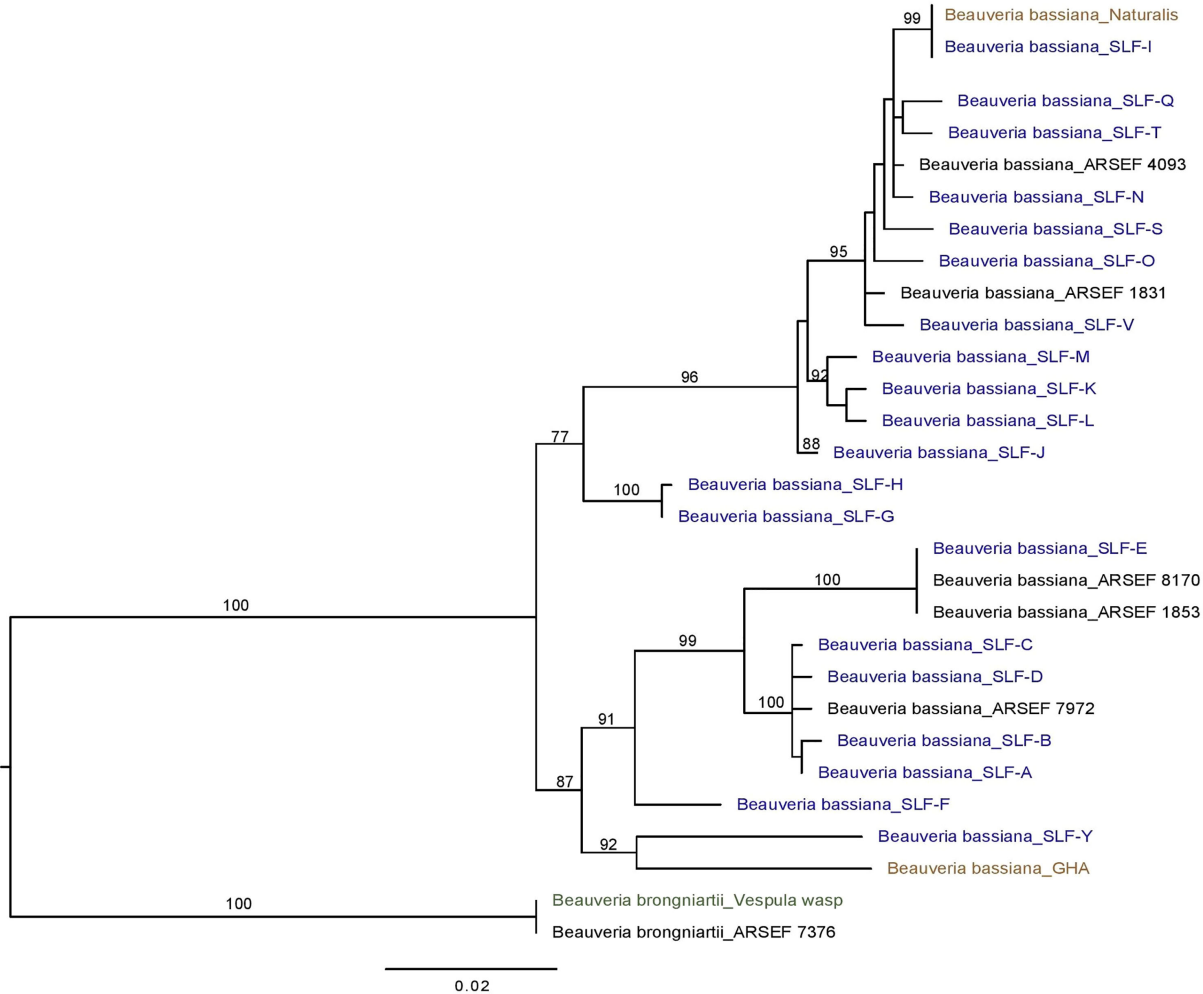
Phylogeny of *B bassiana* associated with the spotted lanternfly (SLF; in blue text), *L. delicatula*. Best tree from Bayesian analysis of the B locus sequence data (alignments of 934 bp). The analysis includes 20 *B bassiana* strains from SLF, the commercial strains GHA and Naturalis (in brown text) and *B brongniartii* as outgroup, including one strain obtained from a yellowjacket (*Vespula* sp.; in green text).

**Figure 4:**
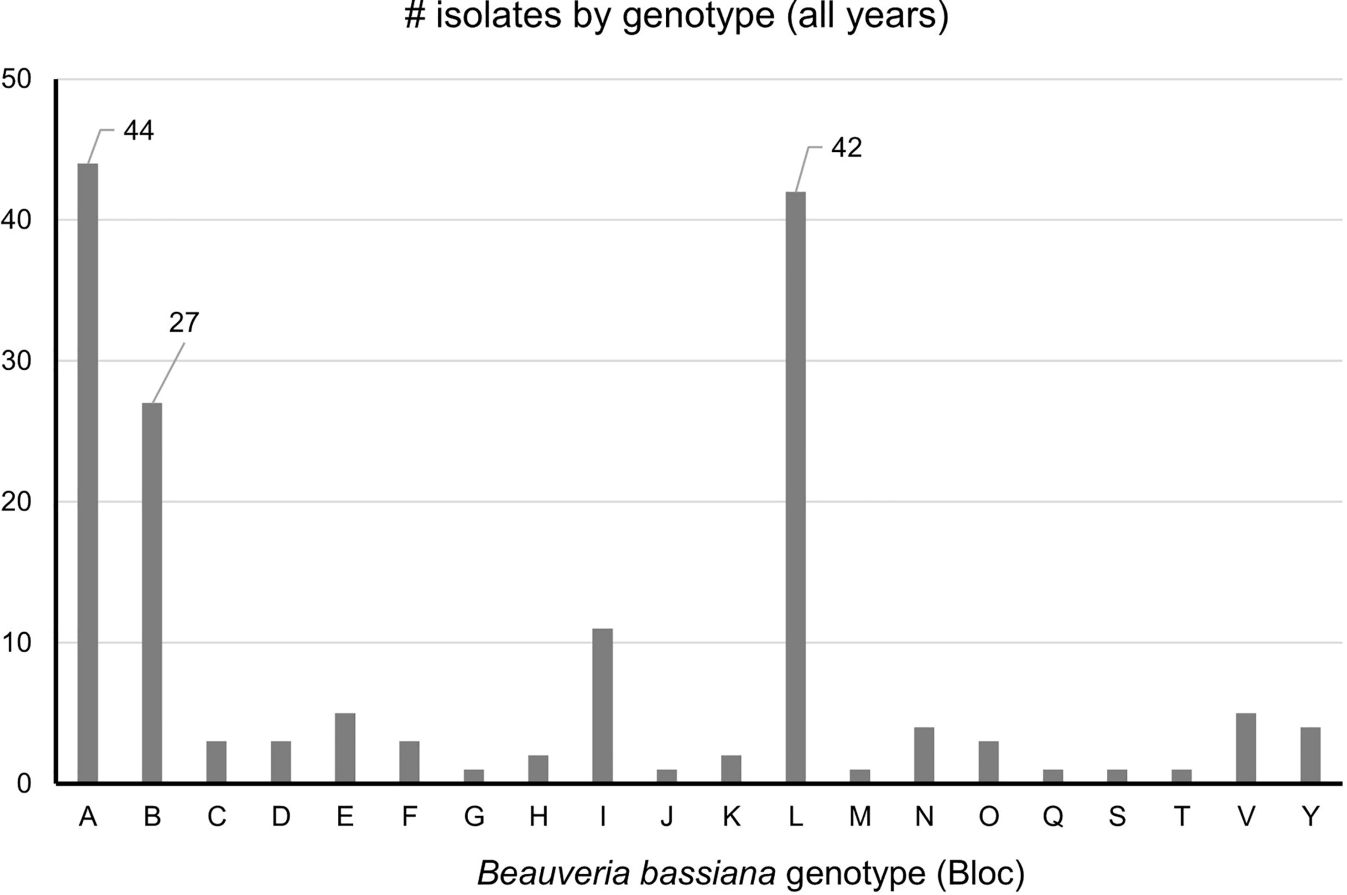
Prevalence of *B bassiana* strains associated with spotted lanternfly adult and nymphs collected in 2017-2020 from Pennsylvania, USA. The most common strains (A, B, and L) have data labels. *n* = 164.

*GHA, commercialized strain used in mycoinsecticides. The two sites in Berks County, Pennsylvania had epizootics in 2018 (same sites marked as *c* and *e* on **Table 1** and **Figure 2**). Strains are based on Bloc sequence data (see **Figures 3**, **4**).

We inoculated 50 ml of a liquid medium (20 g L^-1^ glucose, 1g L^-1^ yeast extract, 5 g L^-1^ KNO_3_, 2.5g L^-1^ KH_2_PO_4_, 150 mg L^-1^ chloramphenicol) with conidia from each agar culture in 100 ml Wheaton bottles, which, loosely capped, were then incubated for 4 days at 26° C. Agitation was provided by a magnetic stir bar, rotating at moderate speed in each bottle. This culture medium produces almost pure blastospore cultures. Duplicate (triplicate in the case of strain GHA) amounts of 100 g flaked barley (Grain Millers, Eden Prairie MN), hydrated to 50% V:W with reverse osmosis water, were autoclaved in vented, plastic, 30 x 20 cm mushroom spawn bags (Mycolabs, Crystal Lake IL) at 122° C for 25 minutes. After cooling, each bag was inoculated with 10 ml liquid culture and sealed. Blastospore concentrations in the inocula ranged from 4-9 x10^7^ ml^-1^. We incubated these bags of solid substrate at 25-26° C for 14 days after which the sporulated barley was transferred to aluminum trays and air dried with gentle laminar air flow over the trays. Drying was complete within 4 days with a final water activity of 0.42-0.45. Conidia were mechanically harvested through stacked 12-mesh and 80-mesh sieves using a table-top automatic powder sifter (Sidasu, Amazon.com). The harvested conidia were then dried to a final water activity of 0.25-0.30 by exposure to silica dessicant in a sealed chamber for 3-4 days. The field-derived strains and commercial strain GHA were grown simultaneously to harvest conidia for bioassays. Yields were calculated based on hemocytometer counts of appropriately diluted spore suspensions in 0.01% Silwet L77 of 100 mg harvested conidial powder and 1 g samples of the spent substrate after conidia had been harvested. Yields were adjusted to conidia Kg^-1^ dry flaked barley. After analyzing the data on conidial production on flaked barley, conidia of each of the higher-yielding isolates of each strain (02-A, 21-L, 58-B) and GHA were used in laboratory bioassays against SLF in June – August 2021.

One to two days before bioassays, we measured the viability of strains by spreading a dilute aqueous conidial suspension on Sabouraud dextrose agar. Germinated conidia were counted at 400× magnification 14–18 h after incubation at 25°C. All strains had 90% or higher viability before the bioassays on SLF.

#### Collecting and rearing *L. delicatula*

For all bioassays, we collected SLF in Pennsylvania and reared them at the Sarkaria Arthropod Research Laboratory at Cornell University, under USDA-APHIS permits (P526-18-02512 and P526P-21-02895). We collected SLF nymphs and adults from the same field site in Sinking Spring, Pennsylvania, as described by Clifton & Hajek (32). Additional details regarding growing *A. altissima* plants, SLF collection, and rearing in the laboratory are also provided in the **Supplementary Materials** for Clifton & Hajek (32). Before bioassays, we reared SLF in 91 cm mesh cages (61 cm L × 61 cm W× 91 cm H; ASIN #B07GN4BWZ7, RESTCLOUD) containing potted *A. altissima*. Cages were held on shelving units in walk-in environmental chambers (10.2 m^2^; 22.5°C:15°C day:night) with a photoperiod of 16:8 [L:D] h and 65% RH. Light ballasts (New Wave T5 48, Sunlight Supply Inc., Vancouver, Washington) with 4 bulbs (F54T5/840, colour temperature 4000 K, Philips Lighting, Eindhoven, Netherlands) were suspended from the tops of shelving units for illumination.

#### Direct spray applications and daily mortality checks

We conducted bioassays with *B. bassiana* against 3^rd^ instar, 4^th^ instar, and adult SLF. We applied field-derived strains and *B. bassiana* strain GHA at the same concentration of 1.0 × 10^7^ conidia mL^-1^ suspended in 0.05% sterile Silwet. We cold anesthetized SLF at 4°C for 8–10 min and transferred to 355 mL cardboard cups (diameter: 9.2 cm, height: 6.4 cm) before spray applications. The middle of the corresponding lid for each cardboard cup was punched out and a 15 ×15 cm piece of polyester tulle fabric (bridal veil mesh; pore size = 4 mm2 (2 mm × 2 mm; W× L); ASIN #B01NAU9OD5) was tightly secured between the lid’s rim and the cup to contain SLF. Cardboard containers were only used once for each replicate spray application and disposed. 1.0 mL of each suspension was applied *via* airbrush. The control treatment was 0.05% Silwet with no *B. bassiana* spores. Containers held 20 nymphs (3^rd^ and 4^th^ instars) or 15 adults (either male or female) during spray applications. After spray applications, we inverted containers on top of newspaper to dry for 10 minutes. Nymphs were transferred to 63 cm mesh cages (24.5 L × 24.5 W× 63 cm H; BugDorm 4M2260, MegaView Science Co., Ltd., Taiwan) containing one potted *A. altissima* plant. We sprayed one container of male adults and one container of female adults separately before they were transferred to same mesh enclosure with potted *A. altissima.* We used larger plants with a 100-cm mesh bag and bamboo support for the adult SLF, which is also described in Clifton & Hajek (32). We sprayed five containers for each life stage and treatment (*n* = 100 nymphs; 75 males; 75 females).

Before spraying SLF with *B. bassiana* suspensions, we prepared 1 cm^2^ squares of water agar that were cut from 150 mm Petri dishes that were prepared in the laboratory. Each agar square was transferred to a smaller 60 mm Petri dish. As we started to spray SLF in each cardboard container, we briefly stopped and sprayed a water agar square for one second before the remainder of the 1.0 ml suspension was applied to SLF. A similar method was used by Poprawski et al. (38) to measure conidia coverage. The lid was placed on the 60 mm Petri dish with the sprayed water agar squares, labelled with the treatment and replicate, and then placed in the refrigerator. Within 48 h of spray applications, we stained the agar squares with lactophenol cotton blue to count conidia and confirm that spray coverage was consistent among treatments. For each water agar square, we scanned 20 random microscope fields at 400×. Counts were averaged and expressed as dosages applied per square millimeter.

We checked SLF daily for 14 days after treatment. We removed SLF that died within 24 h of treatment from cages and excluded them from analysis. Mortality within 24 h was low; for example, 4^th^ instar SLF sprayed with *B. bassiana* averaged 0.65 ± 0.22 dead nymphs 1 day after spraying. During daily mortality checks, we transferred SLF cadavers from each replicate cage to plastic well plates (24 wells per plate; 1.9 cm^2^ surface area per well) held inside sealed plastic food containers (13 × 13 × 5 cm), lined with moistened filter paper, to promote fungal outgrowth and confirm mortality from fungal infections (39). We kept SLF cadavers in these sealed containers for 10–14 days after the time of death to allow for fungal outgrowth. After fungal outgrowth, we covered these well plates with respective lids and stored them in a refrigerator for 20-30 days. The well plates allowed for easy separation of cadavers before we removed *B. bassiana* conidia for quantification (described in next section).

#### Quantification of conidial production on *L. delicatula* cadavers

While carrying out the bioassays and handling SLF that were killed by *B. bassiana* strains, we found apparent differences among strains for conidial production on cadavers (**Supplementary Figure 1**). Previous studies have also quantified *B. bassiana* conidial production on host cadavers as one measurement of epizootic potential (14, 16). For each life stage and treatment, we randomly selected 20 cadavers with *B. bassiana* fungal outgrowth to quantify *B. bassiana* conidial production. Detailed methods for removing and quantifying conidia with 70% ethanol are described in the **Supplementary Materials**. After removing *B. bassiana* conidia from SLF, we dried these cadavers under a fan in a biosafety cabinet for one hour before weighing them on a precision scale for dry body mass (mg). We divided the numbers of total conidia for each cadaver by its dry body mass. While there is less variation in body mass for nymphs, adult SLF can vary a great deal, and adult females are usually heavier than adult males, as noted by Clifton & Hajek (32).

### Data analysis

We calculated mean survival times and standard errors for SLF receiving each treatment based on Kaplan-Meier survival distribution functions using PROC LIFETEST in SAS 9.4 (40). For multiple comparisons of survival times among different treatments, we used the Cox proportional hazards model with PROC PHREG. Contrasts between treatments were conducted using least-square means, adjusted with the Bonferroni correction. For 3-4 instar nymphs, we combined data before we compared survival times (*n* = 200 per treatment). For adult SLF, we combined data for males and females and compared mycoinsecticide treatments. (*n* = 150 per treatment).

For each spray trial on 3^rd^ instars, 4^th^ instars, adult males, and adult females, we compared mean conidial coverage (# per mm^2^ on water agar squares) using a one-way ANOVA with Fisher’s least-significant-difference test, using PROC ANOVA. For counts of conidial production on SLF cadavers, we also used one-way ANOVA and compared total conidia per dry mg of body mass. We checked these data with the Shapiro-Wilk Test (α=0.05) and they did have normal distribution before analysis (41).

## Results

### Identification and diversity

Sequencing of the Bloc region resulted in 931-934 bp of reliable data for 165 isolates, with 812 constant sites and 119 variable characters. One strain (F) had an extra 3 bp (CCC) between positions 527-528 in the Bloc sequence. All *Beauveria* isolates from SLF were identified as *B. bassiana* (**Figure 3**). One isolate from a nontarget yellowjacket wasp was identified as *B. brongniartii* (**Figure 1C**). Bloc sequence data revealed 20 distinct strains among the 164 *B. bassiana* isolates from SLF and non-targets in Pennsylvania, and these strains were grouped into two clades (**Figure 3**).

### Non-targets killed by *Beauveria* spp.

Seven non-target cadavers with fungal outgrowth were collected in 2018-2020. Two of these seven non-targets were yellowjacket wasps infected by *Beauveria* spp. Six non-targets (**Table 4**) were killed by *B. bassiana* strains A, B, D, and V, which were also isolated from SLF. *B. bassiana* strains A, B, and D were placed in one clade while strain V was placed in a separate clade. One isolate of *B. brongniartii* was recovered from a yellowjacket wasp in 2019, but at this time we have not yet recovered this species from SLF.

**Table 4:** Summary of non-target insects killed by *Beauveria* spp.

Site	Date sampled	Non-target	Isolate #	Entomopathogen species	*B. bassiana* Bloc strain
Fleetwood	5/23/2018	Ant	18-434	*Beauveria bassiana*	A
Kutztown University	10/04/2018	Nitidulid beetle	18-376	*Beauveria bassiana*	D
Angora Fruit Farm	10/09/2018	Acalypterate fly	18-356	*Beauveria bassiana*	V
Conrad Road	10/09/2018	Stonefly	18-357	*Beauveria bassiana*	B
Angora Fruit Farm	10/01/2019	Vespid (yellowjacket wasp)	19-508	*Beauveria brongniartii*	N/A (not applicable)
Hill Road	9/15/2020	Pyralid moth	20-030	*Beauveria bassiana*	B
Blandon	10/01/2020	Vespid (yellowjacket wasp)	20-069	*Beauveria bassiana*	B

2018: 4 non-target cadavers infected by *B. bassiana.*

2019: 1 non-target cadaver infected by *B. brongniartii.*

2020: 2 non-target cadavers infected by *B. bassiana.*

### Prevalence of *B. bassiana* strains

Strains A, B, C, L were the only strains recovered from both SLF nymphs and adults. In total, ten *B. bassiana* isolates were recovered from nymphs. Strains A, B, and L were the most prevalent, accounting for 114 isolates out of the total 164 isolates (70%) that were analyzed in this study (**Figure 4**). These dominant strains were found in multiple years for some sites, including Angora Fruit Farm, Blandon, Conrad Road, and Sinking Spring (**Table 5**). Many of the *B. bassiana* isolates sampled from Angora Fruit Farm and Conrad Road in October 2018 came from SLF adults during an epizootic that caused localized population collapses (8).

**Table 5:** The most common *B. bassiana* strains (A, B, and L) based on B locus sequences, associated with spotted lanternfly (SLF) and other hosts in sites in Pennsylvania, USA.

Strain	Sampling Site	County	Year (# samples/total)	Host(s)
A	Angora Fruit Farm	Berks	2018 (12/30); 2019 (1/3)	SLF
	Blandon	Berks	2018 (1/2); 2019 (1/5)	SLF
	Boyertown reservoir	Berks	2017 (1/1)	SLF
	Conrad road residence	Berks	2018 (6/13)	SLF
	Fleetwood residence	Berks	2017 (3/4); 2018 (1/1)	2017: SLF; 2018: ant (1)
	Kutztown University	Berks	2018 (1/7)	SLF
	Leesport	Berks	2019 (3/5)	SLF
	Lilitz residence	Lancaster	2018 (1/1)	SLF
	Penn State Berks	Berks	2018 (1/1)	SLF
	Pottstown Quarry	Montgomery	2018 (2/12)	SLF
	Schuler Road	Berks	2018 (5/18)	SLF
	Sinking Spring	Berks	2019 (4/14)	SLF
B	Angora Fruit Farm	Berks	2018 (1/30); 2020 (1/6)	SLF
	Blandon	Berks	2019 (1/5); 2020 (2/5)	2019: SLF; 2020: SLF (1), yellowjacket wasp (1)
	Conrad road residence	Berks	2018 (4/13); 2020 (6/8)	2018: SLF (3), stonefly (1); 2020: SLF
	Hill road	Berks	2020 (2/3)	SLF (1), pyralid moth (1)
	Kutztown University	Berks	2018 (4/7)	SLF
	Leesport	Berks	2019 (1/5)	SLF
	Schuler Road	Berks	2018 (2/18); 2020 (1/1)	SLF
	Sinking Spring	Berks	2020 (3/6)	SLF
	Treichlers Bridge	Lehigh	2020 (1/1)	SLF
L	Angora Fruit Farm	Berks	2018 (7/30); 2019 (1/3); 2020 (1/6)	SLF
	Blandon	Berks	2018 (1/2); 2019 (2/5); 2020 (2/5)	SLF
	Conrad road residence	Berks	2018 (1/13)	SLF
	Graffa Pond	Lancaster	2020 (6/7)	SLF
	Lancaster Central Park	Lancaster	2020 (1/2)	SLF
	Overlook Park	Lancaster	2020 (5/6)	SLF
	Pottstown Quarry	Montgomery	2018 (3/12)	SLF
	Schuler Road	Berks	2018 (5/18)	SLF
	Schuylkill Center for Environmental Education	Philadelphia	2020 (1/1)	SLF
	Sinking Spring	Berks	2019 (6/14); 2020 (3/6)	SLF

### Bioassays with *B. bassiana* and *L. delicatula*

For all the spray experiments with *B. bassiana* strains, there was no significant difference among treatments for conidia coverage on squares of water agar. Third instar nymphs: no significant differences (F = 1.28, df = 3, 16, *P* = 0.3156), with averages that ranged from 467 to 573 conidia per mm^2^. Fourth instar nymphs: no significant differences (F = 2.39, df = 3, 16, *P* = 0.1068), with averages that ranged from 472 to 563 conidia per mm^2^. Adult males: no significant differences (F = 2.28, df = 3, 16, *P* = 0.1182), with averages that ranged from 525 to 571 conidia per mm^2^. Adult females: no significant differences (F = 0.78, df = 3, 16, *P* = 0.5215), with averages that ranged from 538 to 572 conidia per mm^2^. These data provided confidence that SLF exposed to the *B. bassiana* strains had received similar doses.

**Figure 5:**
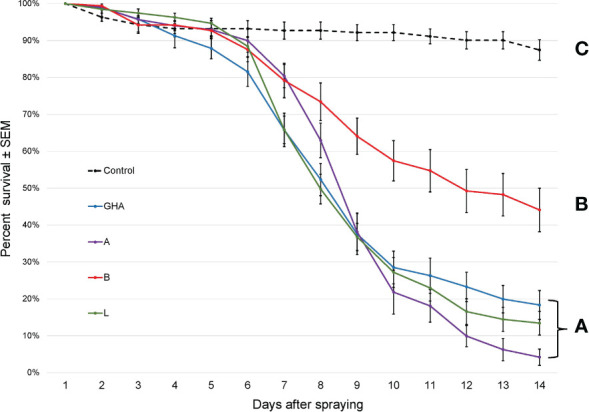
Survival of *L. delicatula* nymphs on potted *A altissima* plants. Data are combined for 3^rd^ and 4^th^ instar nymphs. Lines represent different *B bassiana* strains (control = dotted line). Letters represent significant differences among treatments for survival curves based on Cox proportional hazards.

The survival times of SLF nymphs were significantly different among treatments, (Log-rank χ^2^ = 306.41; df = 4; *P* < 0.0001); those exposed to *B. bassiana* died significantly faster than controls (**Figure 5**). Additionally, *B. bassiana* strain B caused significantly less mortality to SLF nymphs compared to *B. bassiana* strains A, L, and GHA.

**Figure 6:**
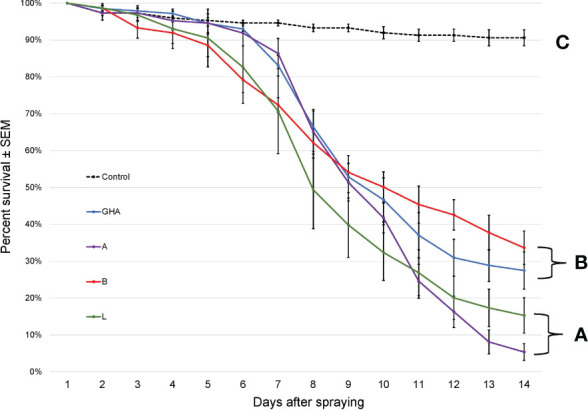
Survival of *L. delicatula* adults on potted *A altissima* plants. Lines represent different *B bassiana* strains (control = dotted line). Letters represent significant differences among treatments for survival curves based on Cox proportional hazards.

The survival times of SLF adults were significantly different among treatments, (Log-rank χ^2^ = 216.68; df = 4; *P* < 0.0001); those exposed to *B. bassiana* died significantly faster than controls (**Figure 6**). Additionally, SLF adults exposed to *B. bassiana* strains A and L died significantly faster than those exposed to strain B and GHA.

**Figure 7:**
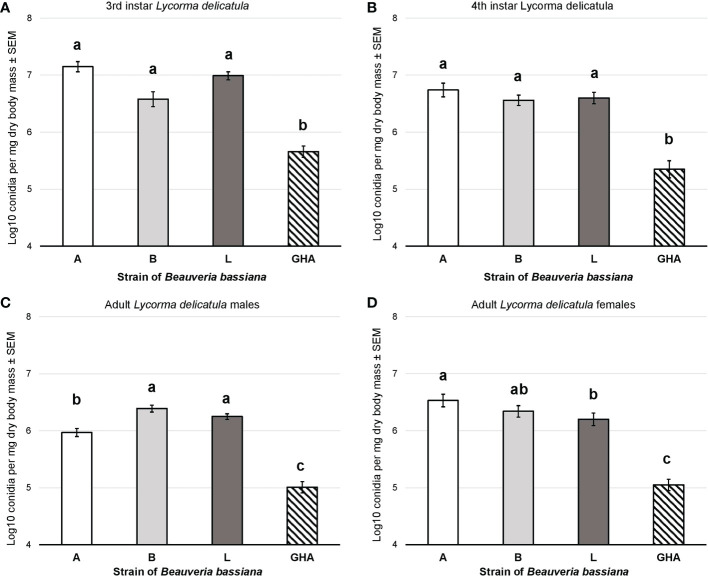
Conidial production of *B bassiana* strains on *L. delicatula* cadavers from experiments on **(A)** third instar nymphs, **(B)** fourth instar nymphs, **(C)** adult males, and **(D)** adult females. Bar shading represent different *B bassiana* strains used in bioassays. Lowercase letters above bars represent significant differences among treatments based on Fisher’s least-significant-difference test.

Conidial production for *B. bassiana* strains A, B, and L was significantly higher than strain GHA for both the 3^rd^ instar nymphs (F = 44.87, df = 3, 75, *P* < 0.0001; **Figure 7A**) and 4^th^ instar nymphs (F = 29.67, df = 3, 76, *P* < 0.0001; **Figure 7B**). Conidial production for *B. bassiana* strains B and L was significantly higher than strain A for adult males, which was significantly higher than GHA (F = 72.94, df = 3, 76, *P* < 0.0001; **Figure 7C**). For adult females, conidial production for strain A was significantly higher than strain L, but strain B was not significantly different compared to both A and L (F = 40.78, df = 3, 76, *P* < 0.0001; **Figure 7D**). All three field-derived *B. bassiana* strains had significantly higher conidial production than GHA for adult females.

## Discussion

All *Beauveria* isolates infecting SLF collected in Pennsylvania from 2017 to 2020 were identified as *B. bassiana*. *Beauveria bassiana* is also known to infect SLF in China (42), where this invasive host is native. *Beauveria bassiana* was also a major entomopathogen infecting invasive scolytines attacking coffee berries in Hawaii (13) and infecting emerald ash borers in Michigan and southwestern Canada (14). This fungal species acts as an entomopathogen but also persists in ecosystems as a plant endophyte, or a saprophyte in the soil (43). As an entomopathogen *B. bassiana* is a generalist, occurring worldwide and known to infect > 200 species of insects across many insect orders (3), although host range is more limited for individual strains (44). Perhaps the breadth in host range of *B. bassiana* as well as life as a saprophyte and endophyte help to explain the natural occurrence of abundant genetic diversity in *B. bassiana*. In our study alone, among *B. bassiana* isolates from SLF in southeastern Pennsylvania we found 20 different strains based on Bloc sequence data. Other studies, each based on one host species, have also documented genetic diversity in *B. bassiana* isolates (13, 14). Our genetic analysis is based on only one locus commonly used for identification of *Beauveria* species (12), but sequencing additional loci or genes could show more genetic differences among these 20 strains.

We hypothesize that the *B. bassiana* isolates from our study are native and their ability as generalists facilitates their switching over to use the relatively new resource constituted by abundant SLF populations. Some of the same *B. bassiana* strains that infected SLF also infected non-targets in this study (**Table 4**), suggesting that these native hosts could constitute some of the sources for the naturally occurring strains. Infection levels by *B. bassiana* will vary over time and space, depending on population densities of susceptible hosts, the fungal titers in the soil and on phylloplanes, and weather (45, 46). Additional sampling methods, e.g., the “*Galleria* bait method” (47) and serial dilutions of soil samples on selective media, could help to better describe the communities of native entomopathogens in these sites that were invaded by SLF.

Three *B. bassiana* strains (A, B, and L) were most prevalent and widespread among field sites in Pennsylvania. *Beauveria bassiana* strain A did not have any 100% matches to *B. bassiana* isolates on GenBank, but it did have high similarity (99-99.8% matches) to Bloc sequences from *B. bassiana* isolates recovered from emerald ash borer populations in Canada (GenBank JN849673.1 and others in PopSet 379045698). Strain L and other strains in the top clade (**Figure 3**) had high similarity to Brazilian strains of *B. bassiana* (ARSEF 4093 and 1831; **Table 2**), and the *B. bassiana* strains in this top clade already have a wide distribution in mainland North America and South America. According to the Bloc sequence data, strain I has a 100% match to *B. bassiana* strain ATCC 74040, which is used in the mycoinsecticide Naturalis^®^ (GenBank KM031766.1), but other loci would need to be sequenced to confirm that strain’s similarity. *Beauveria bassiana* strain ATCC 74040 was originally isolated from the cotton boll weevil*, Anthonomus grandis* (Boheman) (Coleoptera: Curculionidae), in Texas and is known to naturally occur throughout the United States (48). Genetic analysis of *B. bassiana* isolates infecting SLF in other countries is needed to further assess the specificity of these fungal entomopathogens and insect hosts.

In the field studies, we found *B. bassiana* strain A infecting one ant and strain B infecting 3 different non-target insects (**Table 4**). Among the three most collected *B. bassiana* strains, strain L was the only one not found infecting non-targets. Two of the seven non-targets that we found infected by *Beauveria* were yellowjacket wasps (*Vespula* sp. [Hymenoptera: Vespdae]), which were regularly observed foraging for honeydew produced by SLF feeding. We isolated and identified one isolate of *B. brongniartii* from a yellowjacket wasp in 2019 (**Table 4**). *Beauveria brongniartii* has a more restricted host range than *B. bassiana*, principally infecting Coleoptera but also known to infect other insect hosts (12), but to our knowledge this fungus is not associated with SLF or other fulgorids, even though it was collected in the same areas as SLF. It is likely that there is horizontal transmission of *Beauveria* spores between SLF and these hymenopterans, especially since the other vespid had been infected by strain B, one of the most common strains infecting SLF. It should be noted that our sampling of non-targets may not capture the full range of potential non-targets killed by *Beauveria* spp. in these sites. We primarily sampled near *A. altissima* trees, collecting any dead and mycosed non-targets that we could find, often in proximity to dead SLF; otherwise, we did not spend additional time in field sites exclusively looking for non-targets. A variety of insect traps, nets, and other sampling tools are needed to thoroughly describe potential infections of these native non-targets and to find any possible fungus-infected hosts before they are lost to scavengers and weathering.

The laboratory bioassays revealed differences in efficacy, with *B. bassiana* strain B killing significantly fewer nymphs than strains A, L, and GHA, but the bioassays against adults showed that strains A and L had greater efficacy than strains B and GHA. All the field-derived *B. bassiana* strains had significantly greater conidial production on cadavers compared to commercial strain GHA on all SLF life stages that were tested. Wraight et al. (16) found that *B. bassiana* strain HI-25 produced >2.5 times greater numbers of conidia than strain GHA on coffee berry borer cadavers. In our study, we found that strains A, B, and L produced >15 times more conidia than strain GHA on 4^th^ instar SLF (**Figure 7B**), and these results on conidial production suggest these strains have greater epizootic potential than strain GHA. A similar pattern of conidial production was observed for the same *B. bassiana* strains growing on selective medium, with strain GHA having smaller colony growth compared to the field-derived strains (**Supplementary Figure 2**).

More work is needed on these field-derived *B. bassiana* strains to discern any potential for commercialization, in particular relative to the ability to economically mass produce them. If the fungal growth process were scaled up to the larger spawn bags used by industry, it is not yet clear how cost effective it would be to grow these different *B. bassiana* strains even though some strains produced similar if not higher yields than strain GHA on a small scale (**Table 3**). Aside from considerations regarding feasibility of mass production of new *B. bassiana* strains, there would also need to be evaluations of shelf-life, toxicity testing, and other criteria (49). Additional studies are needed to further characterize these *B. bassiana* strains, which would include non-target testing, nutritional requirements for large scale fermentation, and more bioassays to determine virulence to SLF (LC_50_ tests).

In summary, aside from epizootics driven by *B. major* in 2018, *B. bassiana* is the most common entomopathogenic fungus that naturally infects SLF each year in the areas we studied. Surprisingly, among *Beauveria* spp. isolated from SLF, we only found the species *B. bassiana*. Twenty-one *B. bassiana* strains infected SLF, with three being more common. Based on bioassay data, strains A and L are more promising candidates for biological control of SLF, exhibiting similar efficacy to a commercialized strain (GHA) and high epizootic potential due to more abundant conidial production.

## Data availability statement

The datasets presented in this study can be found in online repositories. The names of the repository/repositories and accession number(s) can be found in the article/**Supplementary Material**. Additional raw data supporting the conclusions of this article will be made available by the authors, without undue reservation.

## Author contributions

All authors designed the study. EC and SJ conducted the experiments. All authors analyzed the data and wrote the manuscript. All authors contributed to the article and approved the submitted version.
