# Supplementary material for: Cryptic diversity and virulence of Beauveria bassiana recovered from Lycorma delicatula (spotted lanternfly) in eastern Pennsylvania
Source: Front Insect Sci. 2023 Apr 25;3:1127682. doi: 10.3389/finsc.2023.1127682 (PMC10926454; doi:10.3389/finsc.2023.1127682)

# Supplementary Materials

**Supplementary Table 1**. Conidial yield from *Beauveria bassiana* strains produced on barley flakes with solid state fermentation. Strains with asterisks were used for bioassays against *Lycorma delicatula*.

| **Yield (conidia/Kg substrate)** | | |  |
| --- | --- | --- | --- |
| **Strain** | **Mean** | **S.D.** | **Viability** |
| **LF-02-A*** | 1.08E+13 | 4.39E+12 | 95% |
| **LF-03-B** | 7.10E+12 | 1.81E+11 | 95% |
| **LF-21-L*** | 2.18E+13 | 4.05E+12 | 92% |
| **LF-45-A** | 1.33E+13 | 2.00E+12 | 94% |
| **LF-58-B*** | 9.13E+12 | 2.02E+12 | 91% |
| **LF-67-L** | 1.12E+13 | 1.92E+12 | 94% |
| **GHA*** | 1.29E+13 | 1.43E+12 | 98% |

**Supplementary Methods**

*Removal and quantification of Beauveria bassiana conidia from Lycorma delicatula cadavers*

Nymphs

1. Put a single *Lycorma delicatula* (SLF) nymph with *Beauveria bassiana* conidia into a 1.5 mL centrifuge tube containing 1.0 mL of 70% ethanol. Label the tube with appropriate information about that cadaver (metadata on treatment, date of death, etc.)
2. Agitate the tube on vortexer for 20-30 seconds.
3. Carefully remove the cadaver with fine-tip forceps and move to another tube (can use a 2.0 mL screwcap tube) that has 1.0 ml of 70% ethanol.
4. Put the 1.5 mL tubes into the mini centrifuge. Leave out the second tube for each sample that has the SLF cadaver and residual conidia.
5. Spin the 1.5 mL tubes for 60 s at 10,000 RPM.
6. Pellet of *Beauveria* conidia should be visible at the bottom of the spun tubes.
7. Carefully remove the supernatant from the spun tube while not disturbing the pellet. Can leave a small amount of ethanol at the bottom.
8. Vortex the other tube containing the cadaver for 20-30 seconds. Remove the cadaver dry it on filter paper in a laminar flow hood or biosafety cabinet for one hour.
9. Transfer the remaining ethanol and spores from second tube to the original 1.5 mL tube.
10. Spin the 1.5 mL tubes again for 60 s at 10,000 RPM.
11. Carefully remove the supernatant (again) from the tube while not disturbing the pellet. Can leave a small amount of ethanol at the bottom.
12. Arrange the tubes in a plastic tube rack with the lids open. Place the tube rack inside laminar flow hood or biosafety cabinet with the fan running. Allow ethanol to evaporate for 1 hour (or longer if needed) so that there is minimal liquid in the tubes with conidia pellets.
13. Add 0.5-1.0 mL of 0.05% Silwet to the tubes with the conidia pellets, recording the amount of Silwet that was used (typically using less Silwet when there are less conidia removed).
14. Close the tube lids, organize tubes into a labelled cryovial box and store them in the fridge (assuming they get counted sooner than later). Can store samples in the -20 freezer if they are going to get counted on a later date.

Adults

1. Put a single *Lycorma delicatula* (SLF) adult with *Beauveria bassiana* conidia into a 50 mL centrifuge tube containing 3-5 mL of 70% ethanol. Label the tube with appropriate information about that cadaver (metadata on treatment, date of death, etc.).
2. Agitate the tube on vortexer for 20-30 seconds. Allow the tube to sit for 5 minutes. Agitate again on vortexer for 20-30 seconds.
3. Carefully remove the cadaver with forceps and dry it on filter paper in a laminar flow hood or biosafety cabinet for one hour.
4. Put the 50 mL tubes into a large centrifuge and spin for 2 minutes at 3,000 – 4,000 RPM.
5. Pellet of *Beauveria* conidia should be visible at the bottom of the spun tubes.
6. Carefully remove the supernatant from the spun tube while not disturbing the pellet. Leave enough ethanol in the tube so that the total volume is 2.0 mL or less.
7. Vortex the 50 mL tube with the conidia and <2 mL of ethanol for 30 seconds.
8. Transfer the suspension of ethanol and spores from this tube to 1.5- or 2.0-mL microcentrifuge tube.
9. Spin the microcentrifuge tubes again for 60 s at 10,000 RPM.
10. Carefully remove the supernatant (again) from the tube while not disturbing the pellet. Can leave a small amount of ethanol at the bottom.
11. Arrange the tubes in a plastic tube rack with the lids open. Place the tube rack inside the fume hood or laminar flow hood with the fan running. Allow ethanol to evaporate for 1 hour (or longer if needed) so that there is minimal liquid in the tubes with conidia pellets.
12. Add 0.5-1.0 mL of 0.05% Silwet to the tubes with the conidia pellets, recording the amount of Silwet that was used (typically using less Silwet when there is less conidia removed).
13. Close the tube lids, organize tubes into a labelled cryovial box and store them in the fridge (assuming they get counted sooner than later). Can store samples in the -20 freezer if they are going to get counted on a later date.

**Supplementary Figure 1.** Examples of the typical patterns of *Beauveria bassiana* outgrowth (conidial production) that was observed on *Lycorma delicatula* cadavers. The cadavers in this collage are third instar *L. delicatula* nymphs. Note that *B. bassiana* strain GHA has less conidia production on the cadavers compared to the field-derived strains (2A, 58B, and 21L).


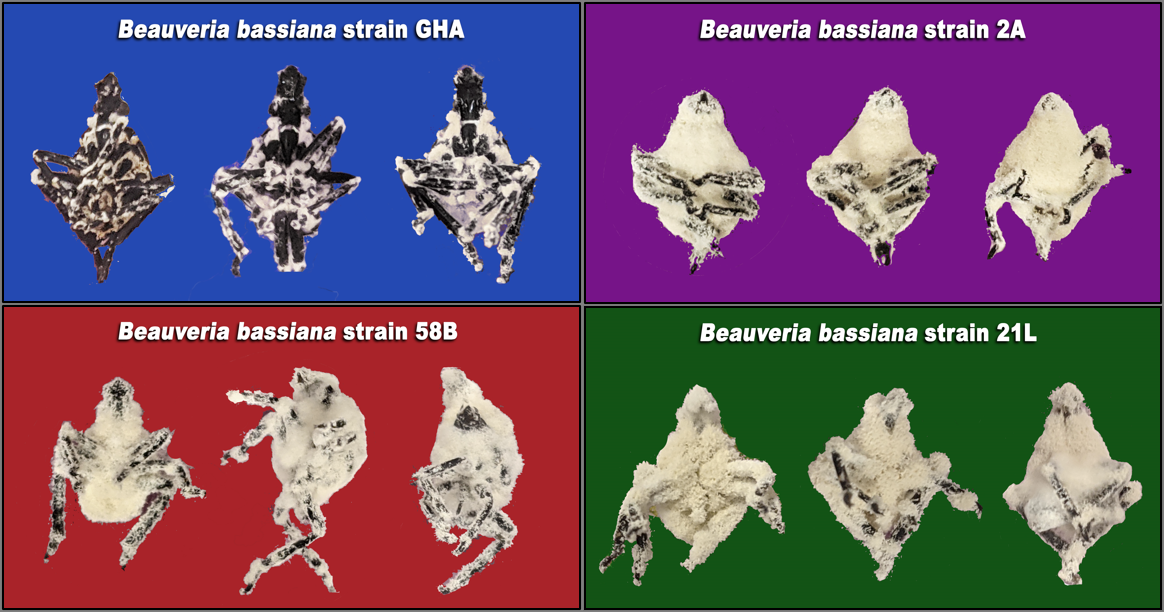


**Supplementary Figure 2.** *Beauveria bassiana* strains used in the *Lycorma delicatula* bioassays. Cultures were grown on selective medium containing crystal violet for 14 days at 22 ºC.


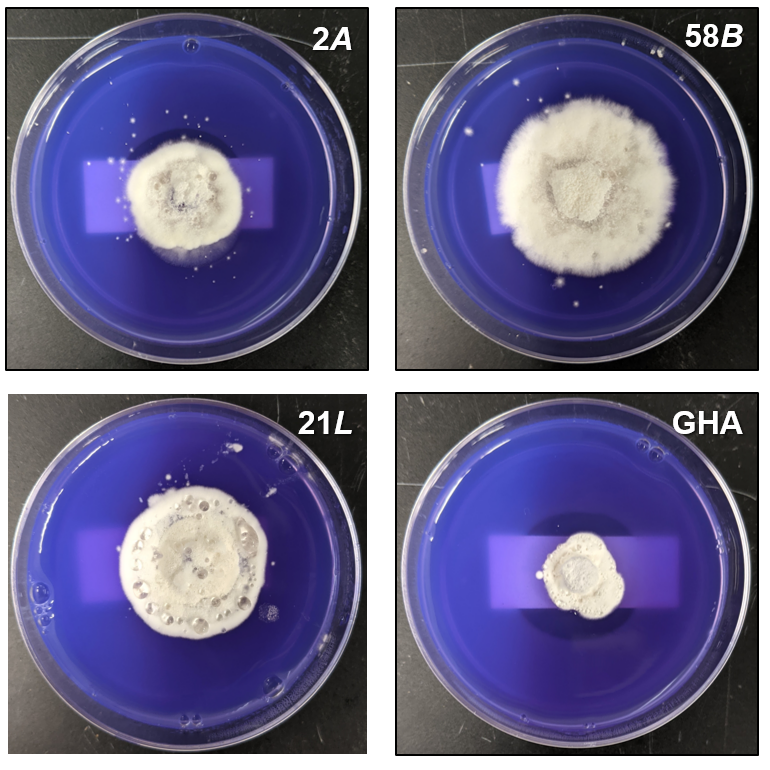

Supplement: Supplementary file 1 [file DataSheet_1.docx]
